# Simultaneous Determination of Five Components of Chaihu-Shugan-San in Beagle Plasma by HPLC-MS/MS and Its Application to a Pharmacokinetic Study after a Single Dose of Chaihu-Shugan-San

**DOI:** 10.1155/2020/8831938

**Published:** 2020-08-19

**Authors:** Yong-liang Zhu, Hui-jun Wang, Hao Xue, Yi Zhang, Qian-shi Cheng, Ling-yun Chen, Xiang-jun Qiu

**Affiliations:** ^1^School of Basic Medicine of Henan University of Science and Technology, Luoyang, China; ^2^School of Public Health and Tropical Medicine, Southern Medical University, Guangzhou, China

## Abstract

Chaihu-shugan-san (CHSGS) has been widely used in China to treat depression and gastrointestinal diseases for thousands of years, but little is known about its pharmacokinetic properties. The purpose of our study is to develop a reliable and sensitive high-performance liquid chromatography-tandem mass spectrometry (HPLC-MS/MS) method to detect five components in beagle plasma and study their pharmacokinetic after oral administration of CHSGS in beagles. An Agilent C18 column (2.1 × 150 mm, 3.5 *μ*m) was used to separate the analytes, and the column temperature was maintained at 40°C. A gradient elution procedure was used with solvent A (acetonitrile) and solvent B (0.1% formic acid, aqueous) as mobile phases. The elution procedure was 60% B—10% B (0–3 min) and 10% B—60% B (3.1–4 min). The flow rate was 0.3 mL/min, and the total measurement time was 4 min. Within the determined range, the standard calibration curves of the five analytes had a satisfactory linear relationship (*r*^2^ ≥ 0.9923). The recovery rate (*n* = 6) of the five analytes was between 85.42% and 90.85%, and the matrix effects (*n* = 6) were between 94.52% and 103.91%. These results show that the validated method could be successfully applied to study the pharmacokinetic in beagles after a single dose of CHSGS.

## 1. Introduction

Chaihu-shugan-san (CHSGS), a classic prescription of traditional Chinese medicine (TCM), is derived from the Book of Jingyue and recorded in the *China Pharmacopoeia* (2015 edition) [1]. CHSGS is famous for its ability to relieve qi stagnation and coordinate gastrointestinal function [2]. With the development of research, CHSGS has been proven to have an excellent effect on depression [3, 4] and functional dyspepsia [5, 6]. CHSGS is a complex prescription consisting of seven components, which is shown in Table 1 [7]. The main chemical components of CHSGS are saponins, flavonoids, phenolic acids, and terpenes. Recent studies have shown that CHSGS has antidepressant, anti-inflammatory, antioxidant, and coordinated gastrointestinal motility [8, 9]. To our knowledge, the flavonoid profile contained in CHSGS is mainly composed of naringin, neohesperidin, hesperidin, paeoniflorin, and liquiritin. Naringin and neohesperidin are two major active ingredients in Zhi-qiao, which are one of the antidepressant mechanisms of CHSGS [10]. Hesperidin, the main active ingredient in Chen-pi, has been proved to coordinate gastrointestinal movements [11]. Paeoniflorin and liquiritin, which are main active ingredients in Bai-shao and Zhi-gan-cao, respectively, are proved to have been used in the treatment of hyperprolactinemia-related disorders [12], in addition to their anti-inflammatory effects [9].

In recent years, a large number of studies have focused on reporting the mechanism of CHSGS [9, 13] and its clinical efficacy [14]. However, to our knowledge, there is no report to elucidate the pharmacokinetic characteristics of CHSGS in vivo. It is known that a pharmacokinetic study of TCM is important to evaluate the rationality and safety of drug prescription [15]. The efficacy of TCM often depends on the synergy between the active ingredients. Therefore, the determination of active ingredients in biological samples is necessary for a pharmacokinetic study of TCM. Although CHSGS has been widely used in clinical application for thousands of years, little is known about its pharmacokinetic properties. Therefore, due to the lack of scientific evidence and research methods, there is no good understanding of the pharmacokinetics of CHSGS in vivo. To improve the development of CHSGS, comprehensive studies of CHSGS are required and a validated bioanalytical method is necessary to support some pharmacokinetic researches.

In this experiment, an efficient, simple, and sensitive HPLC-MS/MS method was established for the first time to simultaneously detect naringin, neohesperidin, hesperidin, paeoniflorin, and liquiritin. Additionally, this validated method was first successfully applied to a pharmacokinetic study of CHSGS in beagles.

## 2. Materials and Methods

### 2.1. Materials

**Figure 1:**
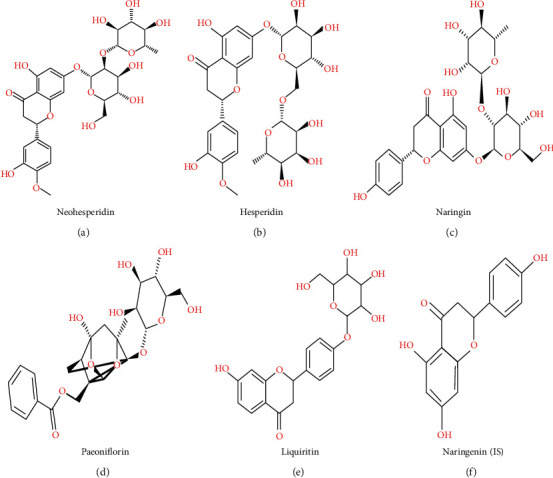
The structure of all analytes and IS.

All analyte standards were purchased from Chengdu Mansite Biotechnology Co., Ltd. (Chendu, China). Naringenin, which was used as internal standard (IS), was bought from Shanghai Yuanye Biotechnology Co., Ltd. (Shanghai, China).  Table 2 shows the specific information of five standards. Meanwhile, the structure of all standards is shown in Figure 1. The deionized water used in the experiment was produced by Milli Q system (Millipore, Bedford, MA, USA). Both acetonitrile and methanol were HPLC grade and were purchased from Merck (Darmstadt, Germany).

### 2.2. Chaihu-Shugan-San Preparation

According to the formula of the *China Pharmacopoeia* (2015 edition), Chai-hu (18 g), Chuan-xiong (15 g), Zhi-qiao (15 g), Chen-pi (18 g), Bai-shao (15 g), Xiang-fu (15 g), and Zhi-gan-cao (9 g) were weighed and air-dried. All materials were bought from Tong Ren Tang Technologies Co., Ltd. (Beijing, China) and had been identified by HPLC-MS/MS method for chemical compositions of plants. In this report, we followed the methods of Li et al. [9] to prepare CHSGS extracts. The raw materials made according to the ratios presented in Table 1 were soaked in 1050 mL ultrapure water (solid/solvent, 1/10) for 30 min, decocted with a large fire until boiling, and then kept boiling for 30 min. After the solution was cooled to room temperature, the solution was filtered using a 0.25 *μ*m microporous membrane. After the solution was concentrated, lyophilized powder was prepared by freeze-drying.

### 2.3. Instrumentation and Conditions

In this report, we used HPLC-MS/MS (Shimadzu, Kyoto, Japan) system to analyze samples. The chromatographic column was an Agilent C18 column (2.1 × 150 mm, 3.5 *μ*m) and the column temperature was 40°C. The gradient elution solutions consisted of mobile phase A (acetonitrile) and mobile phase B (0.1% formic acid aqueous solution). The specific gradient elution procedure was as follows: 0–3 min (60% B—10% B) and 3-4 min (10% B—60% B). The total measurement time was 4 min, the flow rate was 0.3 mL/min, and the injection volume was 10 *μ*L.

Mass spectrometry measurements were performed on a Sciex API 4000 Qtrap MS system equipped with a Turbo Ionspray interface. Samples were analyzed in negative ion mode and monitored in multiple reaction monitoring (MRM) mode. The MS parameters of all analytes are shown in Table 3. The data acquisition and control of the instrument were performed by Analyst 1.5 software.

### 2.4. Standard Solutions, Calibration Standards, and Quality Control (QC) Samples

Stock solutions of all analytes were prepared in the same manner (both at a concentration of 1 mg/mL): accurately weighed 10 mg of the standard and dissolved it in methanol to a constant volume of 10 mL. A working solution for calibration and quality control (QC) was prepared by diluting the stock solution with methanol. A calibration curve standard and QC samples (three concentration levels, low, medium and high) were prepared by adding an appropriate amount of working solution to the blank beagle plasma.  Table 4 shows the specific concentrations of calibration standards and QC samples. The IS working solution 100 ng/mL was also prepared by diluting the stock solution. All solution samples were stored at −20°C.

### 2.5. Preparation of Samples

The samples in this experiment were prepared by ethyl acetate extraction method. After the plasma samples were thawed at room temperature, 100 *μ*L of the plasma sample, 20 *μ*L of IS working solution 100 ng/mL, and 1 mL of ethyl acetate were added into a 2.0 mL Eppendorf tube. The mixed solution was vortexed for 1 min and then was centrifuged at 6,000 ×*g* for 5 min to obtain a supernatant. After the supernatant was dried with nitrogen, acetonitrile-water 40 : 60 was added to reconstitute. 10 *μ*L of the final solution was taken into the HPLC-MS/MS system to analyze.

### 2.6. Method Verification

In this report, we followed the methods of Zhu et al. [16]. Method validation included specificity, linearity, precision, accuracy, recovery, and stability. In this experiment, the HPLC-MS/MS method had been validated according to the US Food and Drug Administration (FDA) guidelines [17].

To verify the specificity of the experimental method, three groups of blood samples were analyzed by HPLC-MS/MS: (a) six individual beagle blank plasma samples; (b) plasma samples added with all analytes and IS; and (c) real plasma samples after giving a single dose of CHSGS. All blood samples were obtained from beagles. Three groups of blood samples were tested for retention time and endogenous interferences.

To evaluate the linearity relationship of the method, a series of concentrations of all analytes were prepared for three consecutive days, each analyte in triplicate. The linear relationship was established using a weighted (1/*x*^2^) least-squares linear regression. The linearity of each standard calibration curve was constructed by plotting the peak area ratio of the all analytes to IS against the nominal concentration of all analytes in plasma. The lower limit of quantitation (LLOQ) was considered as the minimum value of the calibration curve.

The precision and accuracy of this experimental method were verified by repeating QC samples for three consecutive days. Three different concentrations (low, medium, and high) of QC samples were prepared, and each concentration was prepared for six portions (*n* = 6). Quantitative determination of each concentration was performed daily to calculate interday precision, and then the intraday accuracy and standard curve were calculated for three consecutive days. Precision and accuracy were expressed by relative standard deviation (RSD, %) and relative error (RE, %), respectively. RSD = standard deviation/mean × 100%, RE = (average concentration − theoretical average concentration)/theoretical average concentration × 100%. The RSD values were required to be less than 15%, and the RE values were within ±15%.

The recovery of this experiment was calculated by comparing the peak areas obtained in QC samples using ethyl acetate extraction method; the peak areas of the five analytes were obtained using the ethyl acetate extraction method with those obtained from the equal amounts of compounds spiked into the postextraction supernatant at three QC concentration levels (*n* = 6). The above method was also used for the recovery of IS (100 ng/mL). The matrix effects (ME) were calculated by A/B, where A was the peak area of a blank sample spiked with five analytes after extraction and B was the peak area of a standard solution containing equal amounts of the five analytes. The ME of IS (100 ng/mL) were treated in the same way.

In order to test the stability of plasma samples, three different concentrations of QC (*n* = 6) samples were prepared for measurement in 4 different environments (room temperature for 4 h, 4°C for 12 h, −20°C for 4 weeks, and −20°C∼25°C for three freeze-thaw cycles). Short-term stability was calculated by keeping the QC samples at room temperature for 4 h and 4°C for 12 h. Long-term stability was investigated by storing the same QC samples at −20°C in a refrigerator for 4 weeks. The freeze and thaw stability were determined by analyzing the QC samples after three freeze-thaw cycles (−20°C∼25°C). The RSD values were required to be less than 15%, and the RE values were between −15% and 15%.

### 2.7. Pharmacokinetic Study

Six healthy beagles (gender: half male, half female; body weight: 7–9 kg; age: 2-3 years old) were provided by the Laboratory Animal Center of Henan University of Science and Technology (Luoyang, China) and were authorized by the Animal Ethics Committee of Henan University of Science and Technology and were cared in accordance with the National Institutes of Health Guide for the Care and Use of Laboratory Animals. All experimental animals were kept separately in the same conditions (room temperature, 15°C∼28°C; humidity, 35%∼6/0%; light time, 12 h) and fed twice daily without limiting the amount of water. One day before the experiment, the animals were fasted for 12 h but were free to drink water. After giving CHSGS (1 g/kg), 2 mL of blood was collected from the foreleg vein at 0.17, 0.33, 0.5, 0.75, 1, 1.5, 2, 3, 4, and 6 h and was took into heparinized polyethylene tubes. All blood samples were placed in a centrifuge and centrifuged at 10,000 ×*g* for 10 min to obtained 200 *μ*L plasma, which was immediately frozen at −20°C. Using the established method to detect the concentration of five components in plasma, the data of drug concentration of five analytes were dealt with DAS 2.0 and then expressed as the mean ± standard deviation (mean ± SD).

## 3. Results and Discussion

### 3.1. Method Development and Optimization

The IS method is a common method in biological sample analysis. The ideal IS should have the same physical and chemical properties as the analyte. When IS was selected in this experiment, a series of IS, mainly including baicalein, geniposide, paeonol, and naringenin, were analyzed and compared. It was found that when naringenin was used as IS, the reproducibility was good and naringenin did not interfere with the endogenous substances.

Considering the large number of components in the plasma, and each component is different, we used gradient elution for separation. When selecting the mobile phase, we tested methanol-water and acetonitrile-water, respectively. The results showed that when the mobile phase was acetonitrile-water, the resolution between the analytes was higher and the peak shape was better. Additionally, we also tried to add formic acid and acetic acid to the mobile phase to adjust the PH or polarity. The results showed that the addition of formic acid could improve the peak shape and resolution. Meanwhile, when the formic acid concentration was 0.1%, the results were best. Finally, acetonitrile −0.1% formic acid-water was selected as the mobile phase. The use of gradient elution procedure effectively improved the analysis sensitivity and accuracy and significantly reduced analysis time (the total measurement time only needs 4 min).

Common plasma treatment methods include direct dilution method, protein precipitation method, ultrafiltration method, liquid-liquid extraction method, and solid-liquid extraction method [18]. When treating plasma, we compared the use of ethyl acetate extraction, methanol precipitation, and acetonitrile precipitation to process each analyte under medium concentrations, respectively. The results, which were shown in supplementary material, showed that when the ethyl acetate extraction method was used, the sample recovery was higher and the reproducibility was better. Although the methanol precipitation method and acetonitrile precipitation method were simple in operation and short in processing time, they had low recovery rates and poor reproducibility. After the samples were treated by the ethyl acetate extraction method, the LLOQ could be as low as 0.5 ng/mL for naringin and hesperidin and 1 ng/mL for neohesperidin, paeoniflorin, and liquiritin, respectively. Therefore, the method has high sensitivity under the experimental conditions.

### 3.2. Method Validation

**Figure 2:**
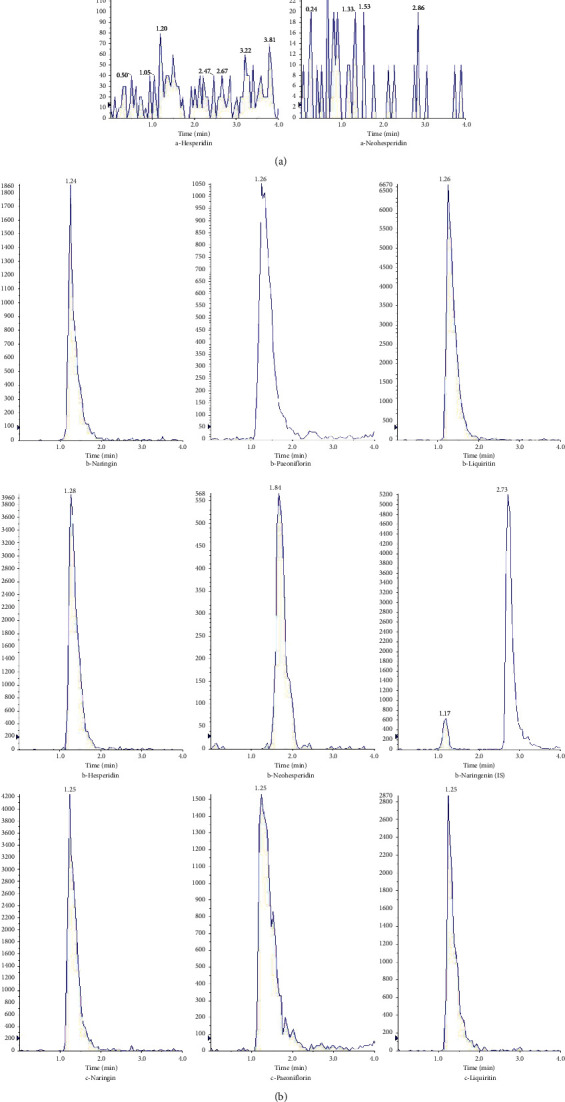
Representative MRM chromatograms: (a) blank plasma; (b) blank plasma spiked with five analytes and IS; (c) real plasma samples after giving a single dose of CHSGS.

Figure 2 shows the results of specificity. The chromatographic peaks of five analytes and IS in plasma were well separated, indicating that the endogenous substances in the beagle plasma did not affect the determination of five analytes and IS. Meanwhile, the retention time of each analyte and IS was 1.24 min for naringin, 1.26 min for paeoniflorin, 1.26 min for liquiritin, 1.28 min for hesperidin, 1.84 min for neohesperidin, and 2.73 min for naringenin (IS), respectively.

The typical regression equations, correlation coefficient, and LLOQ of each analyte are shown in Table 5. *X* represents the plasma concentration and *Y* represents the ratio of the peak area to the peak area of IS. The results showed that the standard calibration curves of the five analytes had a satisfactory linear relationship and all *R*^2^ values were higher than 0.9923.

The precision and accuracy results of each analyte are shown in Table 5. The intraday precision (RSD, %) values of the five analytes were all below 8.80%, while the intraday accuracy (RE, %) values were within ±6.67%. Interday precision (RSD, %) and accuracy (RE, %) values were less than 4.11% and within ±5.11%. Both the RSD and RE values of the intraday and interday met the requirements (RSD < 15% and RE within ±15%), indicating that the method was accurate and reliable.

Table 6 shows that the recoveries ranged from 85.42% to 90.85% at three concentrations (RSD from 2.97% to 8.93%, *n* = 6), and the recovery of IS (100 ng/mL) was 88.96% (RSD = 4.77%, *n* = 6), which indicated that the method was reproducible. The ME of the five analytes were all between 94.52% and 103.91% (RSD from 3.24% to 7.89%, *n* = 6), and the ME of IS was 97.19% (RSD = 3.24%, *n* = 6). The results showed that the ME of plasma did not affect the determination of analytes in this method.

The stability results of this method showed that, at three concentration levels (*n* = 6), the RE of all analytes ranged from −4.71% to 2.97% (RSD ≤ 7.24%) for 4 h at room temperature, −7.29% to 5.23% (RSD ≤ 6.07%) for 24 h at 4°C, −5.30% to 1.94% (RSD ≤ 7.75%) for three freeze-thaw cycles, and −7.00% to 3.64% (RSD ≤ 7.38%), which indicated that there was no significant degradation under the experimental conditions. Therefore, this method established in the experiment was stable.

### 3.3. Pharmacokinetics of Five Analytes

**Figure 3:**
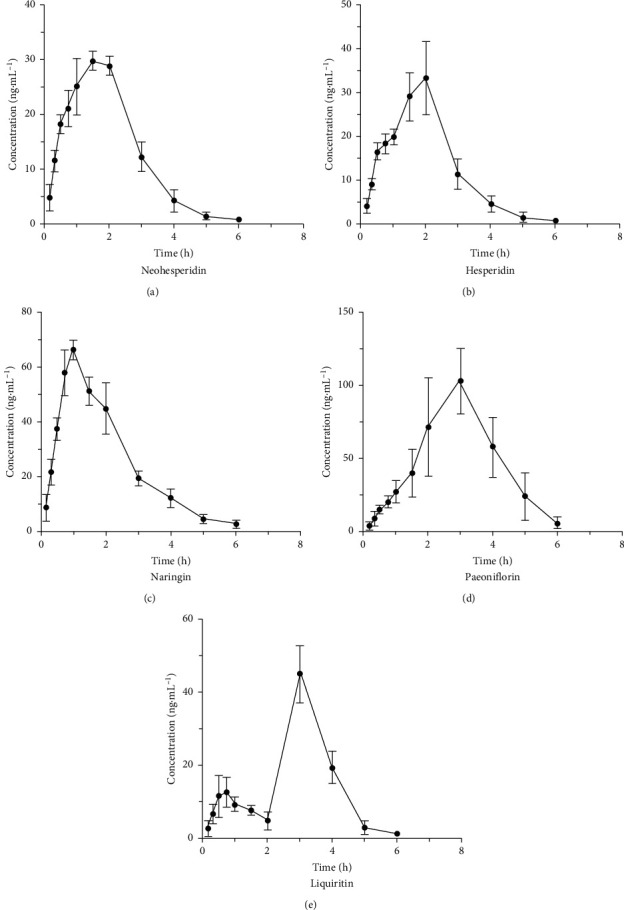
Mean plasma concentration: time curves of five analytes after a single dose of CHSGS (*n* = 6).

The methodological data meet the requirements of FDA, indicating that our method can be used to study the pharmacokinetics of the five analytes. After giving a single dose of CHSGS (1 g/kg), the mean plasma drug concentration-time curves of five analytes in plasma are shown in Figure 3. Additionally, Table 7 shows the main pharmacokinetic parameters of the five analytes, mainly including *t*_1/2_, *C*_max_, *T*_max_, AUC_0−*t*_, AUC_0−∞_, MRT_(0−*t*)_, and MRT_(0−∞)_.

After giving a single dose of CHSGS (1 g/kg), the *T*_max_ values of liquiritin were less than 3 h, and the *t*_1/2_ values were less than 0.55 h, which indicated that liquiritin was absorbed slowly in the blood in beagles but eliminated quickly. Due to the double peak of liquiritin between 2 h and 3 h, the *T*_max_ values of liquiritin were significantly larger than the *T*_max_ values of other analytes. Without double peak, liquiritin might be absorbed faster in beagles. By analyzing the *C*_max_, AUC_(0 − *t*)_, and AUC_(0 − ∞)_ values, the concentrations of the five analytes in plasma were low (all values were less than 297.87 ± 93.01 ng/mL), which might indicate that the pharmacological effect of CHSGS was derived from the superimposed effect after long-term administration. In this experiment, since six beagles were given a single dose of CHSGS, the *C*_max_ values of the analytes were not very high. After a long-term administration, the steady-state plasma concentration of the analytes may be higher. The pharmacokinetic parameters of hesperidin and neohesperidin were extremely close, mainly because hesperidin and neohesperidin are a pair of structural isomers, and their metabolic characteristics should also be similar.

## 4. Conclusion

In this experiment, we established a HPLC-MS/MS method that can simultaneously detect naringin, neohesperidin, hesperidin, paeoniflorin, and liquiritin in beagle plasma. Meanwhile, the method was first successfully applied to a pharmacokinetic study of CHSGS. Our method showed a good linearity for all analytes within acceptable specificity, intra- and interprecision and accuracy.

## Figures and Tables

**Table 1:** The information of components in chaihu-shugan-san (CHSGS).

Botanical name	Herbal name	Chinese name	Acquire from	Ratio (%)
*Bupleurum*	Bupleuri radix	Chai-hu	Root	17.14
*Glycyrrhiza glabra* L.	Glycyrrhizae radix et rhizoma	Zhi-gan-cao	Root and rhizome	8.56
*Paeonia lactiflora* Pall.	Paeoniae radix alba	Bai-shao	Root	14.29
*Citrus reticulata* Blanco	Citri reticulatae pericarpium	Chen-pi	Pericarp	17.14
*Ligusticum chuanxiong* Hort.	Chuanxiong rhizoma	Chuan-xiong	Rhizome	14.29
*Citrus aurantium* L.	Aurantii fructus	Zhi-qiao	Fruit	14.29
*Cyperus rotundus* L.	Cyperi rhizoma	Xiang-fu	Rhizome	14.29

**Table 2:** The specific information of all analytes and IS.

Analytes	Molecular formula	Purity (%)	Batch number
Neohesperidin	C_28_H_34_O_15_	≥98.08	MUST-19040707
Hesperidin	C_28_H_34_O_15_	≥98.46	MUST-19070701
Naringin	C_27_H_32_O_14_	≥98.06	MUST-19050808
Paeoniflorin	C_23_H_28_O_11_	≥99.30	MUST-18032901
Liquiritin	C_21_H_22_O_9_	≥98.50	MUST-18032801
Naringenin (IS)	C_15_H_12_O_5_	≥98.00	L21O10Q100513

**Table 3:** Main analytical parameters of all analytes.

Analytes	Parent ion (*m*/*z*)	Product ion for quantification (m/*z*)	DP^*a*^ (V)	CE^*b*^ (eV)
Neohesperidin	609	301	−138	−65
Hesperidin	609	301	−115	−37
Naringin	579	271	−151	−49
Paeoniflorin	525	449	−66	−20
Liquiritin	417	254	−90	−28

DP, declustering potential; CE, collision energy. ^*a*^Declustering potential. ^*b*^Collision energy.

**Table 4:** The specific concentration of calibration curve standards and QC samples.

Analyte	Concentration range of calibration standards (ng/mL)	Concentration of QC samples (ng/mL)		
		Low	Medium	High
Neohesperidin	0.5, 1, 2.5, 5, 10, 25, 50, 100	1	25	75
Hesperidin	0.5, 1, 2.5, 5, 10, 25, 50, 100	1	25	75
Naringin	1, 2.5, 5, 10, 25, 50, 100, 200	2.5	50	150
Paeoniflorin	1, 2.5, 5, 10, 25, 50, 100, 200	2.5	50	150
Liquiritin	1, 2.5, 5, 10, 25, 50, 100, 200	2.5	50	150

**Table 5:** The typical regression equations, correlation coefficient, and LLOQ in beagle plasma determined by HPLC-MS/MS.

Analyte	Linear range (ng/ml)	Regression equation	*R* ^2^	LLOQ (ng/mL)
Neohesperidin	0.5–100	*y* = 1.79 × 10^−3^*x* + 4.76 × 10^−3^	0.9993	0.5
Hesperidin	0.5–100	*y* = 1.40 × 10^−2^*x* + 2.32 × 10^−2^	0.9953	0.5
Naringin	1–200	*y* = 5.09 × 10^−3^*x* + 8.59 × 10^−3^	0.9980	1
Paeoniflorin	1–200	*y* = 7.54 × 10^−3^*x* + 2.85 × 10^−2^	0.9937	1
Liquiritin	1–200	*y* = 1.57 × 10^−2^*x* + 4.73 × 10^−2^	0.9923	1

**Table 6:** The precision, accuracy, recovery, and matrix effects of five analytes in beagle plasma.

Analytes	Added (ng/mL)	Intraday			Interday			Recovery (%)	ME (%)
		Detected (ng/mL)	RSD (%)	RE (%)	Detected (ng/mL)	RSD (%)	RE (%)		
Neohesperidin	1	0.99 ± 0.05	5.32	−1.50	0.98 ± 0.02	2.35	−1.56	89.97 ± 8.02	99.90 ± 3.62
	25	25.28 ± 1.52	6.02	1.13	25.40 ± 0.85	3.35	1.58	85.42 ± 3.74	102.79 ± 5.37
	75	74.92 ± 3.29	4.39	−0.11	75.47 ± 2.47	3.27	0.63	87.21 ± 5.95	100.66 ± 7.42

Hesperidin	1	0.97 ± 0.03	3.45	−3.00	0.99 ± 0.04	3.90	−1.28	89.09 ± 7.62	98.27 ± 4.14
	25	24.58 ± 1.37	5.59	−1.67	24.89 ± 0.35	1.42	−0.43	90.70 ± 6.24	99.41 ± 7.38
	75	76.28 ± 3.40	4.45	1.70	74.50 ± 2.64	3.55	−0.67	89.42 ± 4.52	100.89 ± 5.56

Naringin	2.5	2.47 ± 0.13	5.29	−1.13	2.49 ± 0.04	1.55	−0.51	88.57 ± 5.90	101.86 ± 7.47
	50	50.67 ± 3.85	7.61	1.33	51.15 ± 2.10	4.11	2.29	86.69 ± 6.47	103.53 ± 5.87
	150	150.65 ± 8.51	5.65	0.44	149.39 ± 2.27	1.52	−0.41	90.85 ± 5.51	103.45 ± 3.98

Paeoniflorin	2.5	2.39 ± 0.17	6.98	−4.53	2.37 ± 0.06	2.68	−5.11	89.37 ± 4.99	103.91 ± 6.70
	50	48.27 ± 3.38	7.01	−3.46	48.44 ± 0.93	1.92	−3.12	87.18 ± 7.05	94.52 ± 5.97
	150	147.59 ± 9.13	6.18	−1.61	151.61 ± 2.90	1.91	1.08	89.27 ± 5.25	100.68 ± 4.65

Liquiritin	2.5	2.33 ± 0.18	7.51	−6.67	2.49 ± 0.04	1.66	−0.29	90.12 ± 6.41	102.03 ± 8.05
	50	49.29 ± 4.34	8.80	−1.41	51.16 ± 1.63	3.19	−1.60	89.82 ± 6.38	100.62 ± 7.64
	150	147.60 ± 5.78	3.91	−1.60	153.97 ± 6.00	3.90	2.64	87.41 ± 5.81	99.29 ± 6.46

**Table 7:** Main pharmacokinetic parameters of five analytes after giving a single dose of CHSGS (*n* = 6).

Parameters	Neohesperidin	Hesperidin	Naringin	Paeoniflorin	Liquiritin
*t*_1/2_ (h)	0.69 ± 0.17	0.71 ± 0.18	0.97 ± 0.12	0.75 ± 0.34	0.55 ± 0.06
*T*_max_ (h)	1.58 ± 0.38	1.75 ± 0.27	0.92 ± 0.13	2.67 ± 0.52	3.00 ± 0.00
MRT_(0−*t*)_ (h)	1.88 ± 0.12	1.95 ± 0.12	1.92 ± 0.08	2.94 ± 0.18	2.91 ± 0.07
MRT_(0−∞)_ (h)	1.93 ± 0.13	2.00 ± 0.15	2.07 ± 0.17	2.97 ± 0.17	2.95 ± 0.07
*C*_max_ (ng/mL)	31.22 ± 2.52	36.53 ± 5.44	67.92 ± 3.00	111.24 ± 17.77	45.00 ± 7.90
AUC_(0−*t*)_ (ng·h/mL)	75.48 ± 7.00	75.01 ± 9.91	150.21 ± 15.18	282.07 ± 69.49	85.37 ± 18.81
AUC_(0−∞)_ (ng·h/mL)	76.29 ± 7.27	76.04 ± 10.35	154.02 ± 16.40	297.87 ± 93.01	86.70 ± 19.04

## Data Availability

The data used to support the findings of this study are available from the corresponding author upon request.

## Supplementary Materials

Supplementary Materials Table S1 summarizes the recovery of five compounds using ethyl acetate extraction, methanol precipitation, and acetonitrile precipitation.
