# Supplementary material for: Simultaneous Determination of Five Components of Chaihu-Shugan-San in Beagle Plasma by HPLC-MS/MS and Its Application to a Pharmacokinetic Study after a Single Dose of Chaihu-Shugan-San
Source: J Anal Methods Chem. 2020 Aug 19;2020:8831938. doi: 10.1155/2020/8831938 (PMC7453237; doi:10.1155/2020/8831938)
Supplement: Supplementary Materials — Table S1 summarizes the recovery of five compounds using ethyl acetate extraction, methanol precipitation, and acetonitrile precipitation. [file 8831938.f1.docx]

**Simultaneous determination of** **five** **components of Chaihu-Shugan-San in beagle plasma by HPLC-MS/MS and its application to a pharmacokinetic study after**

**a single dose of Chaihu-Shugan-San**

Yong-liang Zhu^1^, Hui-jun Wang^1^, Hao Xue^1^, Yi Zhang^1^, Qian-shi Cheng^1^, Ling-yun Chen^2, *^, Xiang-jun Qiu^1, *^

^1^ Medical College of Henan University of Science and Technology, Luoyang, PR China

^2^ School of Public Health and Tropical Medicine, Southern Medical University, Guangzhou, PR China

**SUPPLEMENTARY MATERIALS**

Supplementary Table 1: the recovery of five compounds using ethyl acetate extraction, methanol precipitation and acetonitrile precipitation

| Analytes | Added  (ng/mL) | Treated by ethyl acetate extraction | | Treated by methanol precipitation | | Treated by acetonitrile precipitation | |
| --- | --- | --- | --- | --- | --- | --- | --- |
|  |  | Detected (ng/mL) | RSD (%) | Detected (ng/mL) | RSD (%) | Detected (ng/mL) | RSD  (%) |
| Neohesperidin | 25 | 86.28±5.83 | 6.76 | 70.87±4.20 | 5.93 | 68.66±6.30 | 9.18 |
| Hesperidin | 25 | 93.93±4.98 | 5.3 | 64.30±4.62 | 7.19 | 64.06±4.15 | 6.48 |
| Naringin | 50 | 88.80±4.87 | 5.49 | 75.13±6.53 | 8.70 | 83.00±2.04 | 2.46 |
| Paeoniflorin | 50 | 86.01±2.85 | 3.31 | 67.54±5.69 | 8.42 | 74.52±3.86 | 5.17 |
| Liquiritin | 50 | 85.86±6.84 | 7.96 | 60.69±4.28 | 7.06 | 69.80±3.39 | 4.85 |
